# Rescue Mitral Clip Therapy in Hypertrophic Obstructive Cardiomyopathy with Severe Mitral Regurgitation and Combined Shock

**DOI:** 10.1016/j.cjco.2025.10.015

**Published:** 2025-11-01

**Authors:** Ofir Rabi, Shemy Carasso, Yigal Helviz, Mohammad Karmi, Phillip Levin, Mony Shuvy

**Affiliations:** a Jesselson Integrated Heart Center, Shaare Zedek Medical Center, Faculty of Medicine, The Hebrew University of Jerusalem, Jerusalem, Israel; b Department of Intensive Care, Shaare Zedek Medical Center, Faculty of Medicine, The Hebrew University of Jerusalem, Jerusalem, Israel

**Keywords:** hypertrophic obstructive cardiomyopathy, systolic anterior motion, hemodynamic instability, transcatheter edge-to-edge repair, myosin inhibitor therapy

**We describe a case of MitraClip (Abbott vascular) implantation, in a patient with hypertrophic obstructive cardiomyopathy (HOCM), as a bridge to survival. A 53-year-old woman with severe mitral regurgitation (MR) due to systolic anterior motion (SAM) and a left ventricular outflow tract gradient of 154 mm Hg presented with pneumonia, respiratory failure, and hemodynamic collapse, precluding urgent surgery. Transcatheter edge-to-edge repair using 3 MitraClips significantly reduced the MR, permitting liberation from mechanical ventilation and vasopressors. Subsequent mavacamten therapy led to further stabilization. This case illustrates that use of the MitraClip is a feasible rescue strategy in high-risk HOCM patients with refractory MR.**

Management of HOCM with SAM during acute decompensation is highly challenging. SAM contributes to dynamic left ventricular outflow tract (LVOT) obstruction, severe MR, and worsening heart failure. The relationship between septal hypertrophy, mitral valve abnormalities, and hemodynamic instability necessitates individualized care.^1^ Although septal reduction therapies remain the standard for refractory obstruction, decompensated patients may be unsuitable for surgery. In this context, mitral transcatheter edge-to-edge repair (MTEER) may provide a bridge to stabilization.^2^ We present a case of urgent MitraClip implantation in a patient with decompensated HOCM with SAM.

## Case Presentation

A 53-year-old woman with hypertension and known HOCM was admitted with progressive dyspnea, fever, and cough unresponsive to oral antibiotics. Transthoracic echocardiography 1 year earlier had demonstrated preserved left ventricular (LV) function (LV ejection fraction [LVEF] 60%), asymmetric septal hypertrophy (18 mm), dynamic LVOT obstruction (24 mm Hg at rest, 75 mm Hg with Valsalva), and mild-moderate MR. She reported exertional dyspnea but no orthopnea (New York Heart Association class II). Her therapy included carvedilol, amlodipine, valsartan, aspirin, and atorvastatin.

On admission she was hypoxemic (oxygen saturation 90% on high-flow nasal cannula), hypotensive (blood pressure, 103/69 mm Hg), with bibasilar crackles and diffuse bilateral infiltrates on chest radiography. Laboratory testing showed leukocytosis, anemia, hyponatremia, and elevated C-reactive protein level. She deteriorated rapidly, requiring intubation, mechanical ventilation, and vasopressors. Broad-spectrum antibiotics were administered for presumed pneumonia, but no pathogen has been found.

**Figure 1:**
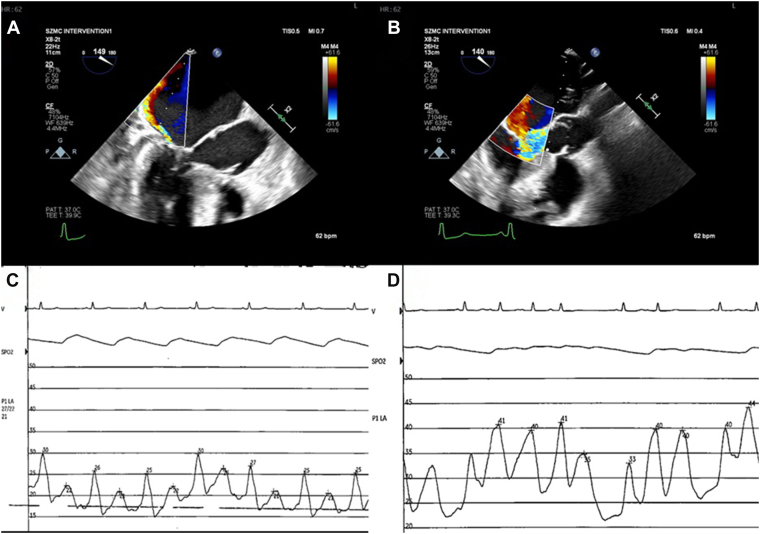
Hemodynamics in systolic anterior motion mitral regurgitation. Transesophageal echocardiographic images and left atrial pressure measurements at the beginning of the procedure: (**A, C**) during high blood pressure vs (**B, D**) low blood pressure. A drop in blood pressure is associated with an increase in left atrial pressures and worsening mitral regurgitation.

Echocardiography in the intensive care unit revealed preserved systolic function, severe septal hypertrophy (24 mm), a maximal LVOT gradient of 154 mm Hg, and severe MR secondary to SAM. Hemodynamic monitoring confirmed elevated filling pressures. Hemodynamic catheterization demonstrated rising left atrial pressure during systemic hypotension—consistent with SAM-related MR (Fig. 1). Despite supportive care, she remained ventilator-dependent, with hemodynamic instability.

**Figure 2:**
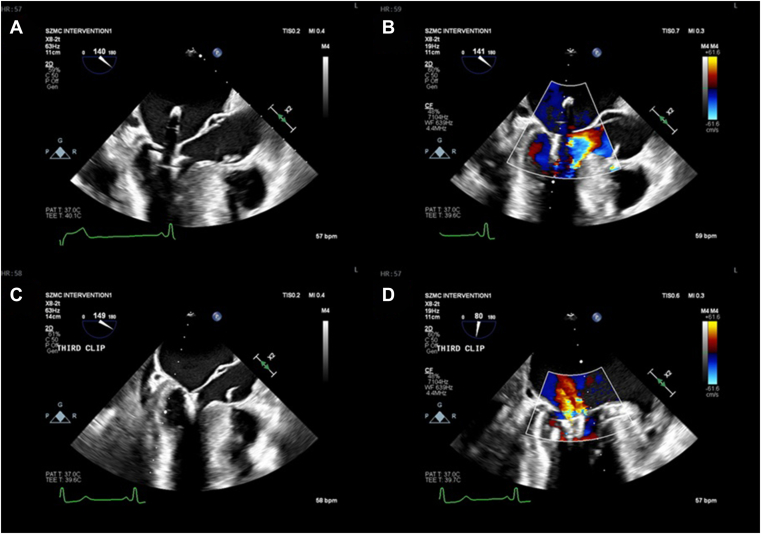
Transesophageal echocardiographic images obtained during and after the procedure. (**A, B**) During clip deployment, the device prevents systolic anterior motion of the mitral valve; (**C, D**) following clip implantation, a reduction in mitral regurgitation is demonstrated.

A multidisciplinary team reviewed several therapeutic options, primarily comparing surgical intervention and MTEER. Given the patient’s critical condition, ultimately MTEER was performed as a life-saving measure. Under transesophageal guidance, 3 clips (XTW and XT, Abbott vascular) were implanted. MR was markedly reduced, pulmonary venous systolic flow was restored, and left atrial V-waves decreased from 50 mm Hg to 25 mm Hg (Fig. 2). No procedural complications occurred. Post-procedure echocardiography demonstrated preserved LV function, a reduced MR grade, and a residual LVOT gradient of 90 mm Hg.

Following MTEER, she was weaned from vasopressors and mechanical ventilation and transferred to the cardiac intensive care unit.

During her stay in the unit, due to persistently elevated LVOT gradients of up to 160 mm Hg despite beta-blocker and calcium-channel-blocker therapy, mavacamten 5 mg daily was initiated during hospitalization. Follow-up echocardiography revealed no decline in LV systolic function, and the patient demonstrated clinical improvement without supplemental oxygen requirements and was discharged.

At a cardiology clinic follow-up 1-month postdischarge, the patient reported clinical improvement, with functional status consistent with New York Heart Association class I-II. Transthoracic echocardiography demonstrated preserved LV systolic function (LVEF 50%-54%), an interventricular septal thickness of 20 mm, and a peak LVOT gradient of 29 mm Hg, with no change in Valsalva maneuver. Following prior MTEER, moderate-to-severe mitral regurgitation persisted, with a mean transmitral gradient of 7 mm Hg. The patient was maintained on propranolol and mavacamten, under which she remained clinically stable. The management plan included continuation of the current regimen with close surveillance. In view of her young age, future septal myectomy with concomitant mitral valve replacement will be considered as a therapeutic alternative.

## Discussion

We report a patient with HOCM and SAM presenting with combined cardiogenic and septic shock—likely triggered by pneumonia on the background of significant LVOT obstruction—who was successfully stabilized with MTEER. The current literature on MTEER in HOCM remains limited to case reports.^2^, ^3^, ^4^ To our knowledge, this case is the first documented successful use of MTEER in an acutely ill HOCM patient in whom the shock was not secondary to a procedural complication.

This case highlights the therapeutic dilemma of managing acute decompensation in HOCM with SAM. Although septal myectomy remains the standard for symptomatic patients, surgery may be unfeasible in unstable settings.^2^ Alcohol septal ablation is another nonsurgical option for symptomatic HOCM, but its outcomes are highly operator-dependent and are best when it is performed at experienced, high-volume hypertrophic cardiomyopathy centres.^5^^,^^6^ Its use in cardiogenic shock has not been documented. In this context, MTEER can provide hemodynamic stabilization, mitigate MR, and facilitate recovery.^2^^,^^3^ In our case, although alcohol septal ablation was considered in a multidisciplinary discussion, it was deferred owing to limited institutional experience with it, and its delayed therapeutic effect, whereas MTEER offered immediate hemodynamic benefit.

In our patient, the procedure was technically challenging due to marked septal thickening and it inherently limits future options to valve replacement. Given the lack of alternatives and hemodynamic instability with failed ventilator weaning, MTEER was performed as a bridge to recovery rather than definitive therapy. The intervention stabilized blood pressure and enabled extubation; however, the limited MR improvement on follow-up echocardiography likely reflects the patient’s significant hypertrophy, rendering her a suboptimal MTEER candidate. Notably, interventricular septum measurements differed between the baseline external study and the in-hospital transthoracic echocardiography, likely due to acute illness–related LV changes and inter-centre variability.

However, even after this significant improvement, the case remained challenging in terms of symptomatic relief. As is well established, the recommended medical management for patients with HOCM consists of beta-blockers and calcium-channel blockers. Cardiac myosin inhibitors, such as mavacamten, are generally reserved for use only after failure of standard therapy.^7^ Moreover, they are generally avoided in the acute setting, because initiation of therapy may precipitate a decline in cardiac function.^6^

Considering the inadequate response to standard therapy and the ongoing inability to achieve clinical stability for discharge, a decision was made to initiate mavacamten during hospitalization, with close monitoring of cardiac function. No deterioration in LVEF was observed, and treatment resulted in significant clinical improvement, enabling discharge for continued outpatient follow-up.

## Conclusions

This case illustrates the complexity of managing HOCM patients with SAM who experience decompensation and, as a result, or for other reasons, are not candidates for surgical intervention. The case demonstrates how the use of MTEER may serve as a therapeutic option in such patients, providing hemodynamic stabilization and acting as a bridge to definitive treatment.

**Novel Teaching Points**

• MitraClip (MTEER) can serve as a feasible rescue therapy in patients with HOCM and severe SAM-related mitral regurgitation when surgery is contraindicated or is not immediately feasible.
• MTEER may provide rapid hemodynamic stabilization, in the setting of cardiogenic or mixed shock.
• Severe septal hypertrophy may limit complete MR resolution, yet partial correction can still yield meaningful clinical recovery and allow transition to definitive therapy.
• Sequential use of mavacamten following MTEER can further reduce LVOT gradients and sustain hemodynamic improvement in selected HOCM patients.
